# Alignment-free prediction of cross-reactivity in influenza A (H3N2) anticipates antigenic drift

**DOI:** 10.1371/journal.pcbi.1014628

**Published:** 2026-08-11

**Authors:** Alpha Forna, Lambodhar Damodaran, Christian E. Gunning, Parnian Rahimi, Aarya Venkat, Natarajan Kannan, Rebecca Kondor, Justin Bahl, Pejman Rohani, John M. Drake

**Affiliations:** 1 Odum School of Ecology, University of Georgia, Athens, Georgia, United States of America; 2 Center for the Ecology of Infectious Diseases, University of Georgia, Athens, Georgia, United States of America; 3 Department of Epidemiology and Biostatistics, College of Public Health, University of Georgia, Athens, Georgia, United States of America; 4 Department of Pathobiology, School of Veterinary Medicine, University of Pennsylvania, Philadelphia, Pennsylvania, United States of America; 5 Institute of Bioinformatics, University of Georgia, Athens, Georgia, United States of America; 6 Center for Influenza Disease & Emergence Research (CIDER), Athens, Georgia, United States of America; 7 Centers for Disease Control and Prevention, Atlanta, Georgia, United States of America; 8 Department of Infectious Diseases, University of Georgia, Athens, Georgia, United States of America; University of Hong Kong, HONG KONG

## Abstract

Since its introduction in 1968, Influenza A (H3N2) has undergone continuous antigenic evolution, necessitating frequent vaccine updates. To predict antigenicity and characterize antigenic drift without multiple sequence alignments, we present *FluEmbed*, a computational framework that leverages protein language models. *FluEmbed* accurately quantified the antigenic impact of viral evolution from RNA sequences, achieving strong predictive performance against hemagglutination inhibition (HI) assay titers (Spearman correlation: *ρ* = 0.67–0.80). *FluEmbed* also outperformed sequence-distance baselines (e.g., Hamming and BLOSUM62) and phylogenetic tree-based models that require sequence alignment. Using this model, we conducted *in-silico* mutagenesis experiments to identify site/amino acid combinations that differentially impacted antigenicity. To systematically investigate how specific mutations influence immune escape, we defined two classes of mutations: ‘constrained’, where only the most likely amino acid changes at historically mutation-prone sites were considered (thereby limiting the mutation space) and ‘unconstrained’, where all possible substitutions were allowed, providing a full exploration of potential antigenic shifts. Constrained mutations often confer limited antigenic changes, whereas unconstrained mutations exhibit greater escape potential, particularly outside the dominant viral lineages. Notably, 3C.2a was the only major lineage in which constrained and unconstrained mutations showed no significant difference (*p* ≈ 0.95), suggesting ongoing intra-clade competition rather than inter-lineage antigenic replacement. By enabling rapid, alignment-free antigenic prediction directly from sequence data, *FluEmbed* could complement traditional HI assays in real-time influenza surveillance and inform vaccine strain selection decisions.

## Author summary

Influenza A (H3N2) viruses have evolved rapidly, necessitating frequent vaccine updates. Computational tools that can rapidly predict antigenic changes from sequence data alone could accelerate vaccine strain selection and reduce reliance on time-consuming laboratory assays. We introduced *FluEmbed*, a computational framework that leverages protein language modelling to predict antigenicity from H3N2 HA sequences. Our approach not only accurately predicts antigenic relationships but also enables *in silico* mutagenesis studies to assess potential evolutionary pathways — *FluEmbed* predicts antigenicity computationally without requiring laboratory assays for each variant. We showed that, while constrained mutations (i.e., mutations limited to frequently observed substitutions at key sites) may impose functional limits on viral evolution, unconstrained mutations (i.e., mutations allowing any amino acid at those key sites) can explore a broader spectrum of antigenic changes. Thus, unconstrained mutations are more likely to enable significant immune escape, which could contribute to future vaccine mismatches. By integrating antigenic surveillance with predictive modelling, *FluEmbed* represents a significant advancement in computational virology, with implications for vaccine design and pandemic preparedness efforts.

## Introduction

Influenza A (H3N2) infections cause significant global morbidity and mortality, necessitating continuous surveillance, virus characterization and frequent seasonal vaccine updates [1,2]. The virus has undergone rapid antigenic evolution, primarily through mutations in the hemagglutinin (HA) protein, a viral surface protein that facilitates immune escape [3]. Understanding and predicting these antigenic changes are critical for vaccine strain selection and public health preparedness [4]. Traditional assay-based approaches, such as hemagglutination inhibition (HI), remain the gold standard for measuring antigenic similarity but are time-consuming, labor-intensive, and limited in throughput [5–8]. HI titers are typically used as a measure of antigenic distance between test and reference strains that is comparable across different antisera. Furthermore, these assays require experimental validation of every likely antigenic jumps, making real-time monitoring challenging [9].

To address these challenges, computational models have been developed to predict antigenicity using nucleotide and protein sequence data [10–12]. Early models relied on multiple sequence alignments and physicochemical descriptors [13–15]; however, these methods have been constrained by their dependence on predefined features [16] and limited generalizability to novel strains [17–19]. More recently, deep learning and protein language models (PLMs) have emerged as powerful tools for encoding protein sequences in high-dimensional space and capturing complex evolutionary patterns without the need for multiple sequence alignments [20–22].

Here, we introduce *FluEmbed*, an alignment-free framework for the antigenic prediction of influenza A (H3N2). Unlike traditional sequence-based methods, *FluEmbed* leverages a previously trained PLM to generate contextual sequence embeddings, preserving both antigenic and structural constraints (we employ Meta’s evolutionary-scale modelling (ESM) PLM [23]). By integrating historical HI assay data with high-dimensional sequence embeddings, we demonstrated that *FluEmbed* accurately predicts antigenic relationships and provides a scalable, data-driven approach for antigenic characterization. To better understand the evolutionary dynamics driving antigenic drift, we developed an *in silico* mutagenesis strategy that distinguishes between ‘constrained’ and ‘unconstrained’ mutations. Constrained mutations are limited to a biologically plausible subset of amino acid substitutions at evolutionarily conserved sites, whereas unconstrained mutations permit all possible amino acid changes at these sites. This reduction in antigenic space enabled us to systematically investigate how different mutation types influence immune escape and vaccine mismatch risk.

By bridging structurally aware computational modelling with ongoing antigenic surveillance, *FluEmbed* represents a significant advance in influenza monitoring and vaccine strain selection. This framework not only enables real-time antigenic prediction but also provides insights into viral evolutionary trajectories, including evolutionary vaccine design, by integrating both past trends and future changes, offering a valuable tool for pandemic preparedness and public health decision-making.

## Results

### Temporal dynamics of HI titers and test-antiserum virus pairs

**Fig 1:**
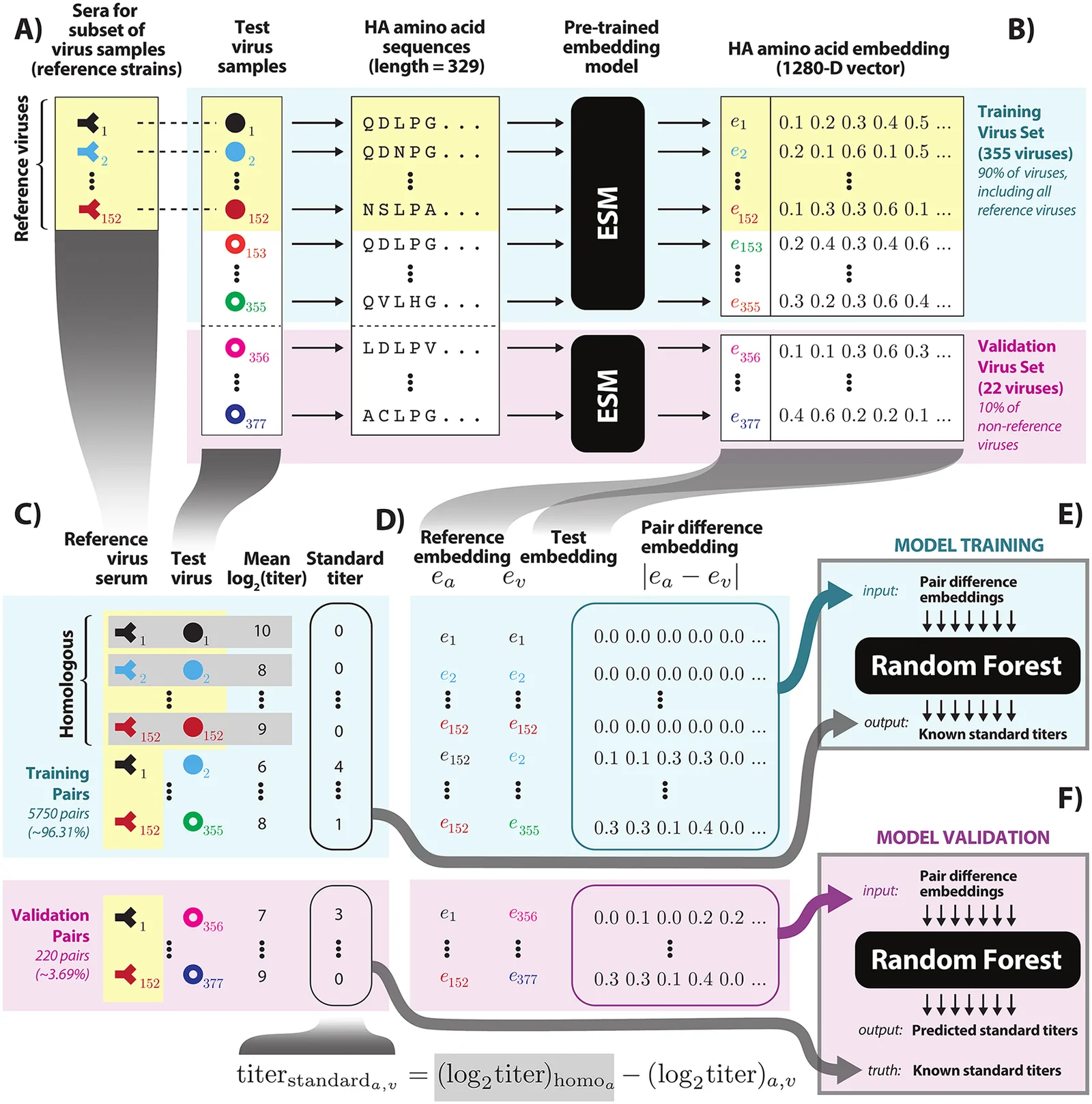
*FluEmbed* is a computational workflow for predicting antigenic interactions between influenza A (H3N2) viruses using HA1 sequence data mapped to standardized hemagglutination inhibition (HI). HA1 is a subunit of the hemagglutinin protein, a viral surface protein that attaches to host cells. Standardized HI titers represent normalized measurements from HI assays that quantify the antigenic relationships between influenza viruses. Lower standardized titers indicate greater antigenic distance between the reference antiserum and the test virus, reflecting reduced antibody recognition due to mutations in antigenic sites that enable immune escape. Training and validation of *FluEmbed* were accomplished in six steps. (A) *FluEmbed* was evaluated using input data comprising the HA amino acid sequences (length = 329) of 5970 virus isolates, representing 355 unique strains. These sequences were processed using a pre-trained ESM embedding model to produce 1 × 1280-dimensional embedding vectors for each virus. (B) Dataset partitioning: The data were divided into training (355 viruses, 90% of the total) and validation (22 viruses, 10% of the non-reference) sets (see Fig A in S1 Appendix for details on data preprocessing and partitioning). (C) Pairwise comparison: Reference virus antiserum and test virus pairs were created using the associated mean ${log}_{\text{2}}$ titer) and standard titer values from hemagglutination inhibition assays. (D) Embedding difference calculation: Pair difference embeddings were computed to measure the difference between each test embedding and all the reference embeddings. (E) Model training: A Random Forest was trained using pair difference embeddings as the input and known standard titers as the output for 5750 training pairs (~96.31% of data). (F) Model validation: The trained model was applied to 220 validation pairs (approximately 3.69% of the data) to predict the standard titers compared to known values.

**Fig 2:**
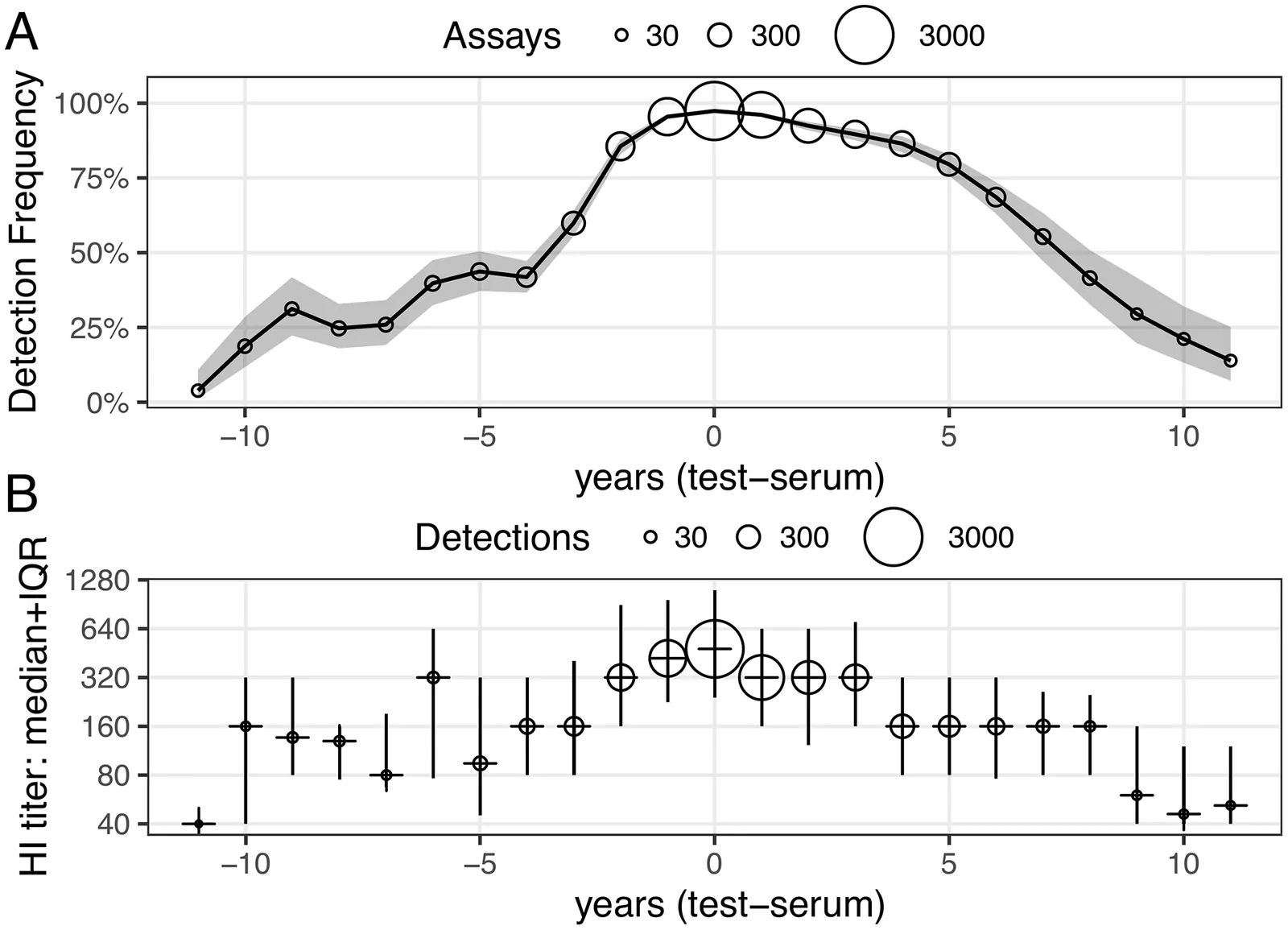
Temporal relationship between test and antiserum virus pairs in the dataset shows the progressive decline in antigenic cross-reactivity over time. (A) Detection (i.e., assays with detectable inhibition) frequency of test-antiserum pairs as a function of the time difference (Δ years) between the two viruses showed that the time difference between most tested pairs was less than 5 years, with the detection frequency dropping rapidly outside this time window. (B) Median HI titers (with interquartile range, conditioned on detection) plotted against Δ years, demonstrating a decline in antibody reactivity as temporal distance increased. Overall, these results show that inhibition drops rapidly with increasing time difference between the test and the reference antiserum.

The *FluEmbed* workflow data inputs and algorithms used in the analysis are described in (Fig 1). We applied the algorithm to test and antiserum virus pairs for a period of 43 years, from 1968 to 2011, as illustrated in Fig 2. Fig 2A shows the detection frequency (i.e., detectable HI) of test-antiserum pairs as a function of the time difference between the first occurrence of each (Δ years). Most HI tests conducted between test-antiserum pairs (circle size) were collected within five years of each other, with the detection frequency dropping rapidly at larger lags. Fig 2B shows how HI titers decline as the temporal distance between the test and antiserum viruses increases, reflecting the accumulation of antigenic drift over time. These results establish the range of antigenic relationships that our framework can capture and emphasize the importance of accounting for temporal dynamics when analyzing antigenic evolution. This context frames our subsequent analyses, which explore constrained and unconstrained mutational pathways to map the antigenic space and predict immune escape potential.

### Sequence embeddings effectively represent antigenic relationships

**Fig 3:**
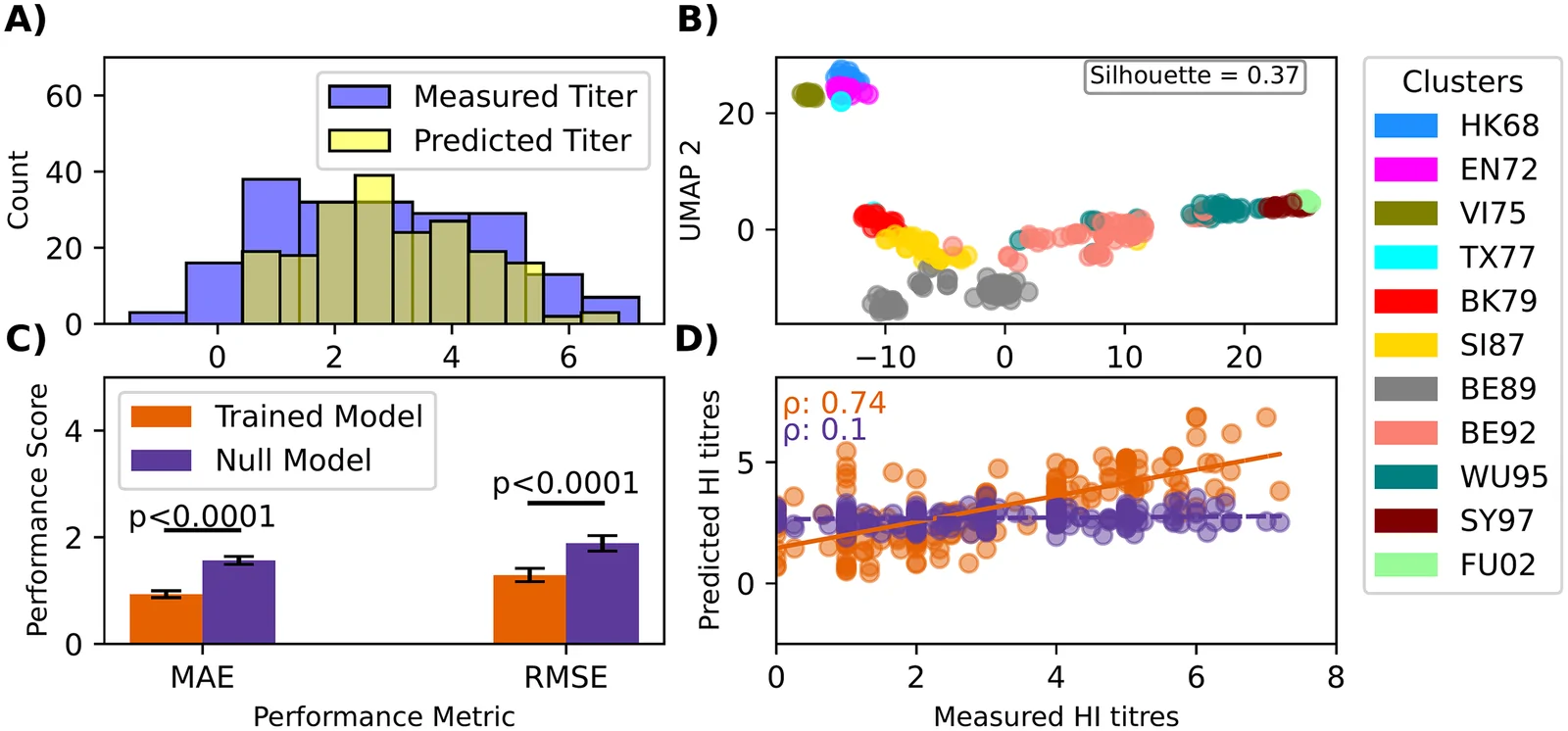
*FluEmbed* accurately predicts influenza A (H3N2) antigenicity using sequence embedding. (A) Distribution of measured (blue) and predicted (green) standardized ${log}_{\text{2}}$ HI titers. (B) UMAP projection of ESM [23] embeddings colored by known antigenic clusters, showing that the encoded space captures serological relationships. (C) Significantly lower mean absolute error (MAE) and root mean squared error (RMSE) for *FluEmbed* than for the null model (non-overlapping confidence intervals, p < 0.0001). (D) High correlation (Spearman’s *ρ* = 0.74) between *FluEmbed* predicted and observed titers, with no correlation (r = 0.1) for the null model.

To generate HA sequence covariates, the ESM PLM [23] was used to create 1280-dimensional embeddings for each virus sequence. These ESM embeddings encode model-predicted, context-dependent information about the protein structure without using traditional sequence alignment. Specifically, each virus and its corresponding antiserum (*e.g.*, the ancestral HK68 strain) were represented by 1280-dimensional embedding from ESM. We then computed the element-wise absolute difference between these vectors, yielding a new 1280-dimensional, unitless vector that quantifies the antigenic distance. These virus-antisera difference embeddings were used as covariates in a random forest model to predict the standardized HI score for influenza. We employed UMAP [24] to reduce the dimensionality of the data from Smith *et al.* (2004) [6] to two dimensions, allowing us to visualize the embedding space. The resulting plot (Fig 3B) revealed that the embeddings were grouped according to antigenic type, with each influenza A (H3N2) strain forming a separate cluster. This outcome validates our methodology by showing that our computational approach independently replicates known antigenic structures without being explicitly programmed to do so. Thus, the embeddings were confirmed to capture antigenically relevant information and are appropriate covariates for mapping the HA sequence to HI titers.

### *FluEmbed* accurately predicts influenza A(H3N2) antigenicity from sequence

*FluEmbed* was trained on a curated dataset of 5,807 HI assays involving 355 unique viruses and antisera raised against 152 unique reference strains [8]. Model tuning and validation were performed through cross-validation to optimize prediction accuracy. Fig 3A shows that *FluEmbed* reproduced the distribution of experimentally measured standardized HI titers on the held-out test data. We adopted a stratified sampling approach to assess *FluEmbed*'s capacity to predict standardized HI titers for novel virus-reference virus pairs. We first identified all unique test virus strains from our database of virus-reference virus pairs, excluding any test viruses that appeared as reference viruses. We then randomly selected 10% to constitute our held-out test data along with their corresponding reference virus pairs, ensuring that the selected test viruses appeared exclusively in the held-out test set. The remaining 90% of virus reference pairs formed the training set. This strategy ensures that *FluEmbed* is evaluated using novel test virus strains that mimic real-world scenarios of emerging influenza variants. Quantitative evaluation demonstrated a high correlation (Spearman's *ρ* = 0.74, *p* < 0.0001) between the predicted and measured titers across the range of assay values (Fig 3 panel D). In contrast, as designed, a null model based on sequences with randomized amino acid assignments performed poorly (*r* = 0.10, *p* < 0.0001; Fig 3 panel D), highlighting the importance of evolutionary constraints [25]. The trained model demonstrated superior performance, with a lower mean absolute error (MAE) than the null model (non-overlapping CIs, *p* < 0.0001) (Fig 3, panel C). *FluEmbed* achieved a numerically lower root-mean-squared error (RMSE) than the null model (Fig 3 panel C). Thus, both the absolute and squared error metrics indicate the superiority of data-driven *FluEmbed* over a random null model. Finally, *FluEmbed* generally exhibited low error rates, with most predictions falling within two-fold dilutions of observed measurements.

*FluEmbed* sequence embeddings achieved superior predictions relative to physicochemical properties across all metrics (Table 1). This includes higher correlation (0.74 vs 0.28, Spearman *ρ*) and lower errors (0.93 vs 1.52 MAE). To further contextualize these results, we benchmarked *FluEmbed* against classical sequence-distance baselines (Hamming, BLOSUM62, Grantham), raw ESM-2 cosine similarity, and the phylogenetic tree-based model of Neher et al. (2016), all evaluated on the same held-out test set (S2 Appendix). *FluEmbed* (ρ = 0.74) substantially outperformed all tested approaches, including the Neher model (ρ = 0.65), which requires a pre-computed sequence alignment and phylogenetic tree. These performance gains demonstrate that protein language model embeddings capture complex evolutionary and structural constraints related to influenza antigenic drift that are not accessible through sequence similarity, physicochemical descriptors, or phylogenetic distance alone.

**Table 1:** *FluEmbed* embeddings improved influenza antigenicity prediction over physicochemical features (hydrophobicity, isoelectric point, Boman’s index, electric charge, and instability index). Compared with physicochemical descriptors, *FluEmbed* with ESM embeddings achieved higher accuracy, demonstrating that sequence representations better capture complex evolutionary constraints.

Model	*FluEmbed*	
Trained covariates	ESM2 Embeddings	Physicochemical Properties
Spearman correlation	0.74 (0.67-0.80)	0.28 (0.14-0.40)
MAE	0.93 (0.80-1.06)	1.52 (1.31-1.74)
RMSE	1.29 (1.11-1.47)	1.87 (1.61-2.13)

### *FluEmbed* reveals temporal heterogeneity in influenza antigenic evolution

**Fig 4:**
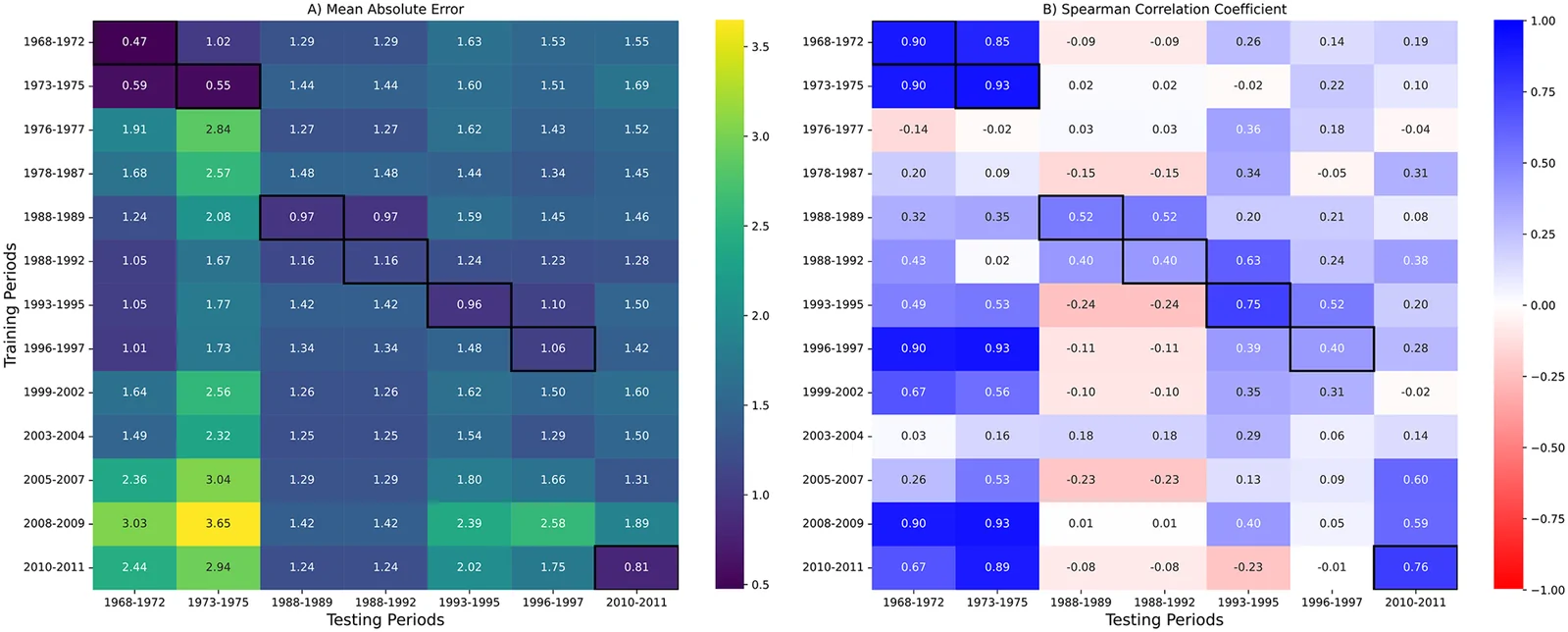
*FluEmbed* reveals temporal heterogeneity (i.e., test periods aligned with the emergence of specific antigenic clusters) in the antigenic evolution of influenza A (H3N2). Model performance was evaluated using (A) the mean absolute error (MAE) and (B) Spearman correlation coefficients between predicted and measured HI titers across different training and testing periods. Diagonal entries represent matched training test periods and show the highest error (yellow) and correlation (blue), with performance deteriorating for out-of-range testing. Notably, in the bottom-left triangle, where more recent training samples predict past test samples, we frequently observed a high MAE and negative correlation.

Fig 4 shows the temporal variation in the ability of *FluEmbed* to predict influenza antigenicity across different training and testing periods. Here we employed testing periods that were aligned with the previously described H3N2 antigenic clusters, such as the HK68 cluster (1968–1972) [6]. This temporal segmentation allowed for biologically relevant analysis, evaluation of *FluEmbed* performance across distinct antigenic epochs, and assessment of its predictive capabilities during viral evolutionary transitions. Compared with the null model, the model exhibited better performance, as indicated by its lower MAE (Fig 4A) and higher Spearman correlation coefficients (Fig 4B) when the training and testing data overlapped. However, the predictive accuracy declined as the time lag between training and testing increased. For the 1968–1972 period, MAE reached its minimum and correlation peaked when tested on contemporary data, but performance deteriorated when applied to future epochs. Similar trends were observed for other periods: optimal results were obtained when training and testing periods overlap temporally, with predictable performance degradation when predicting antigenically distant epochs. Notably, Fig 4 shows forward-in-time prediction (training on earlier data to predict later data), demonstrating that *FluEmbed* generalizes to future antigenic contexts, albeit with increasing error as the temporal gap widens. The 2010–2011 model showed the poorest generalization, particularly when predicting earlier epochs, suggesting that this period encompasses accelerated antigenic drift or represents a distinct evolutionary regime. This pattern is consistent with the known diversification of H3N2 lineages in this period, though we note that reduced generalization could also reflect model limitations at the boundaries of the temporal range. These findings highlight the temporal specificity of antigenic determinants. Global models that aggregate data across all time periods may obscure the distinct impacts of specific amino acid substitutions that drive antigenicity within specific time periods.

We conducted sensitivity analyses to assess the robustness and reproducibility of *FluEmbed* (S2 Appendix). These analyses confirmed: (i) reproducibility of the full pipeline across independent runs with near-identical cross-period performance heatmaps and amino acid importance rankings (S2 Appendix, Figs A–B); (ii) stability of Random Forest performance across multiple random seeds, with most periods showing minimal sensitivity to seed choice (S2 Appendix, Fig L); (iii) improved evaluation stability through stratified temporal sampling that ensures balanced representation across years within each train-test period (S2 Appendix, Figs N–O); (iv) robustness of results to alternative embedding pooling strategies, with classification token pooling (CLS) producing comparable error patterns to mean pooling we used for our main analysis (i.e., the mean pooling approach we are reporting in this manuscript) (S2 Appendix, Section 7); and (v) superior performance relative to the alignment-dependent Neher et al. (2016) tree model, with *FluEmbed* achieving Spearman ρ = 0.727 versus ρ = 0.654 and lower prediction errors across all metrics (S2 Appendix, Section 8).

### *FluEmbed* identifies period-specific substitutions driving influenza antigenic evolution

**Fig 5:**
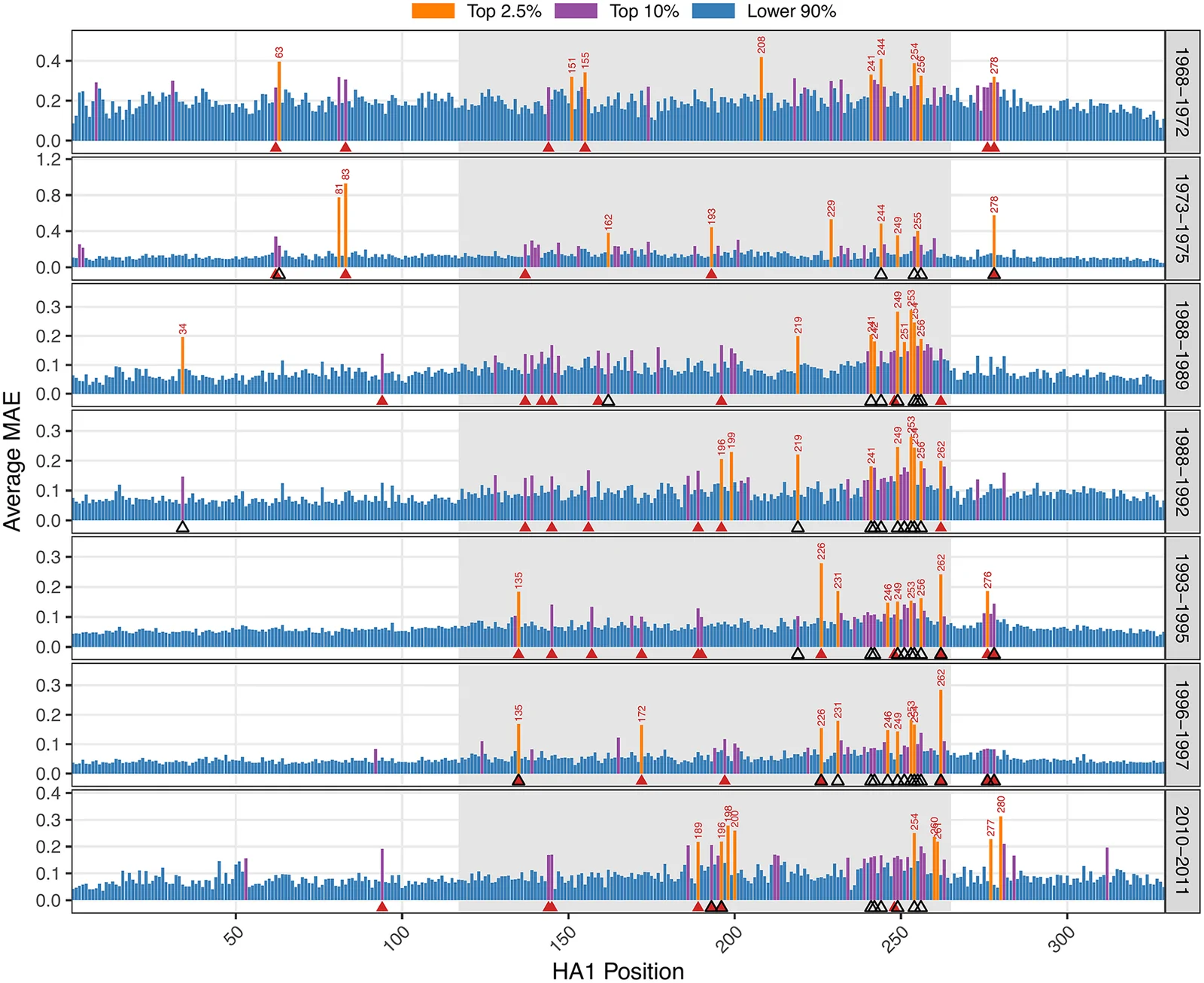
*FluEmbed* identifies period-specific substitutions driving influenza A(H3N2) antigenic evolution. The relative impact of amino acid substitutions within each test period (rows) was ranked (color), with key residues labelled in the top 2.5% of important residues. The y-axis shows the change in average mean absolute error (ΔMAE) when a given residue is deleted, relative to the unmodified sequence. The positions previously identified as antigenic determinants are marked by open black triangles, whereas those appearing across multiple periods are indicated by red triangles. Residues marked with red triangles and black outlines represent sites that persisted throughout the experimental period and were previously identified in the literature.

*FluEmbed* pinpointed key substitutions contributing to influenza antigenic drift within specific periods (Fig 5). Here, ‘specific periods’ correspond to the matching test-training time periods in our data (equivalent to the diagonal in Fig 4). The impact of amino acid changes on the model accuracy was measured by the MAE change with/without a given residue. Here, we highlight the top 2.5% of the positions (labelled residues). Many top-ranked sites, such as 135, 145, 155, 156, 158, 159, 189, 197, 226, and 262 (marked with triangles), were previously identified as antigenic determinants [11,26]. Some residues, such as 241, 242, 246, 252, 254, 256, 260, 261, and 262 (indicated by red triangles), persistently appeared across multiple test periods, suggesting their sustained importance in shaping antigenic trajectories. Other positions exhibited period-specific prominence, reflecting the shifting genetic context of influenza evolution. Overall, the temporal patterns of antigenic determinants revealed by *FluEmbed* align with known molecular and epidemiological evidence [26–28], underscoring our model's capacity to capture the dynamic nature of influenza antigenicity.

### *In Silico* mutagenesis reveals functional constraints on antigenic evolution

Mutations were classified as ‘constrained’ when only the most likely amino acid changes at historically mutation-prone sites were considered, thereby limiting the mutation space, whereas ‘unconstrained’ mutations allowed all possible substitutions at these sites, providing a full exploration of potential antigenic shifts. Constrained mutations often confer limited antigenic changes, whereas unconstrained variants exhibit greater escape potential, particularly outside dominant viral lineages.

**Fig 6:**
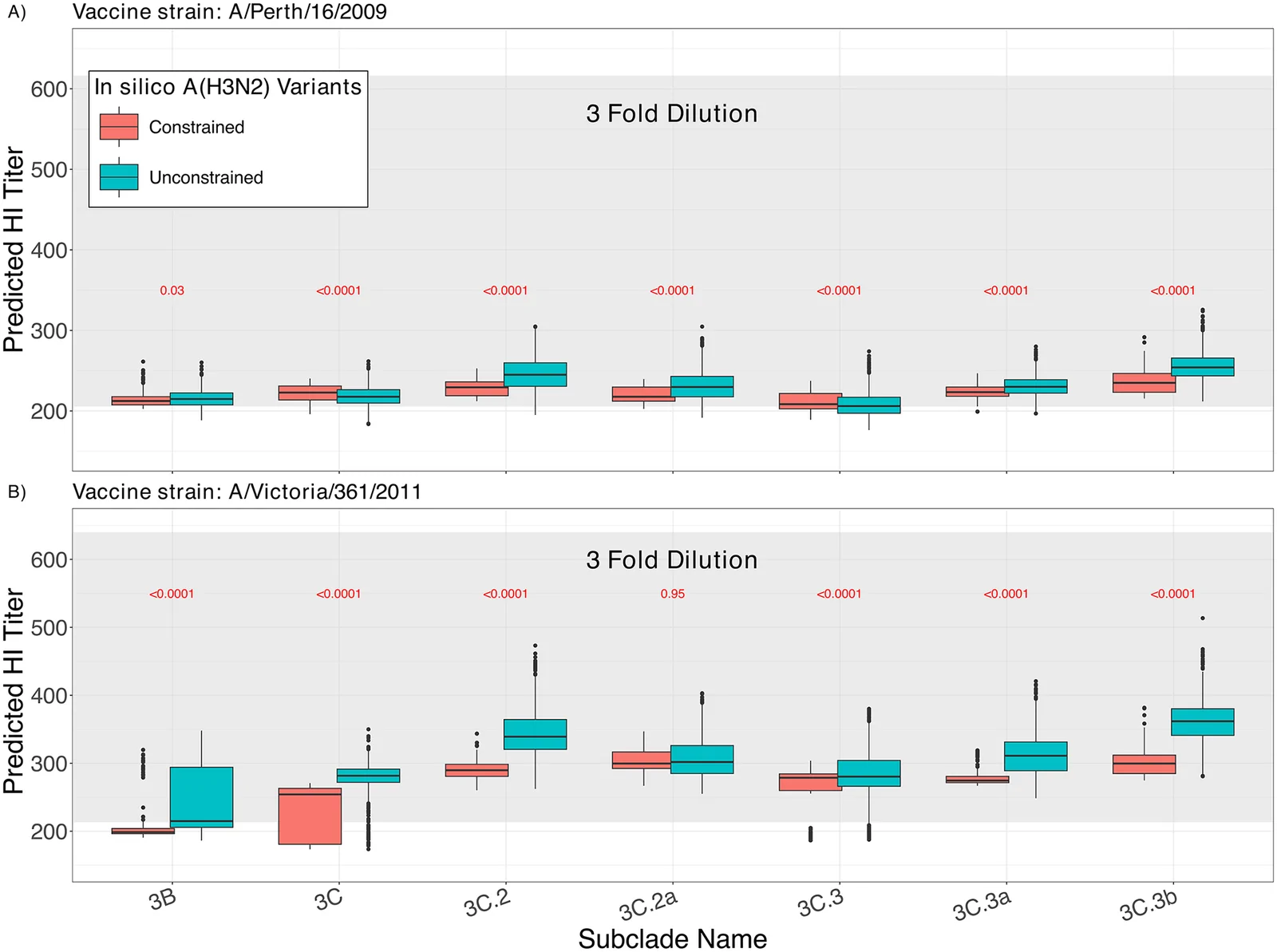
*FluEmbed* reveals differential antigenic impacts of constrained and unconstrained mutations across influenza A(H3N2) subclades. Constrained mutations consider only historically likely amino acid changes at mutation-prone sites, whereas unconstrained mutations allow all possible substitutions. Standardized predicted HI titers represent normalized measurements, where lower values indicate a greater antigenic distance, reflecting reduced antibody recognition due to immune-evading mutations. (A) For the A/Perth/16/2009 and (B) A/Victoria/361/2011 vaccine strains, constrained mutations exhibited a smaller antigenic impact on average, indicating that the resulting variants generally had higher HI titers (closer to the wild-type, indicating less antigenic change). In contrast, the unconstrained mutations showed a wider range of antigenic outcomes. Although their HI titers were higher on average than those of constrained mutations, some unconstrained mutations led to very large titer drops (major antigenic changes), giving this category a broader overall distribution. Notably, in (B), the 3C.2a clade was the only lineage where constrained and unconstrained mutations did not differ significantly (p ≈ 0.95) in HI titer, suggesting that antigenic drift in 3C.2a occurs through finer-scale changes within the clade rather than through big jumps. Note: All p-values are corrected for multiple comparisons using the Benjamini-Hochberg FDR procedure.

Fig 6 illustrates the antigenic impact of constrained and unconstrained mutations across the H3N2 subclades. *FluEmbed* predicted that constrained variants (with substitutions restricted to the most common mutations at historically high-frequency sites) exhibited a more restricted antigenic range than unconstrained variants (which allow any amino acid at those key sites). For the A/Perth/16/2009 and A/Victoria/361/2011 vaccine strains, constrained mutations yielded significantly lower average HI titers (indicating larger antigenic changes). In contrast, unconstrained mutations generally resulted in higher average HI titers (smaller antigenic changes) but with a much broader distribution of outcomes. In other words, most unconstrained mutations caused minimal antigenic changes, yet a few produced very large drops in the HI titer. This broader range of HI titers among unconstrained variants reflects a greater potential for immune escape, although their typical effect is less pronounced than that of constrained mutations. These findings underscore the evolutionary trade-offs that shape viral antigenicity. Functional constraints limit the extent to which mutations can drive antigenic change, on average, whereas unconstrained sites allow occasional large antigenic jumps despite typically smaller changes. Thus, functional constraints can act as a brake on immune escape, even though unconstrained mutations provide opportunities for broader escape outcomes when they occur. This highlights the potential of targeting conserved functional sites during vaccine design to mitigate the antigenic drift.

Clade 3C.2a was the only lineage in which constrained and unconstrained mutations did not differ significantly (Fig 6B, *p* ≈ 0.95), suggesting that antigenic evolution within 3C.2a was not strongly shaped by unconstrained mutations, unlike in other clades, where antigenic escape drives divergence. Instead, the observed antigenic changes appear to be fine-scale adjustments within 3C.2a rather than major shifts leading to new dominant lineages. This pattern is consistent with recent influenza trends, in which 3C.2a-derived viruses remain dominant across multiple seasons [29]. We note that this analysis is based on in silico mutagenesis of 3C.2a consensus sequences using mutation frequencies from *Nextclade*, rather than direct HI measurements from 3C.2a-era strains (which post-date our 1968–2011 training data). The results should therefore be interpreted as a prospective prediction of *FluEmbed*’s utility for characterizing emerging lineages, pending validation with contemporary titer data.

## Discussion

This study demonstrates how protein language models (PLMs) [25] can address the longstanding challenges in influenza surveillance and evolutionary forecasting*.* The modern era has led to rapid and widespread collection of pathogen genome data; however, reliable mapping of protein sequences to biological phenotypes remains an area of active research. With *FluEmbed*, we leveraged modern advances in PLMs that have been previously trained on evolutionary-scale datasets. Our model offers a flexible and extensible framework for scalable, sequence-based quantification of influenza antigenic phenotypes from limited labelled data [30]. Integrating *FluEmbed* into existing influenza surveillance frameworks (e.g., WHO’s Global Influenza Surveillance and Response System (GISRS)) could provide real-time antigenic assessments to aid vaccine strain selection. Using *FluEmbed*, which offers high-throughput antigenic prediction for rapid assessment alongside traditional HI assays, public health agencies can accelerate the early detection of emerging antigenic variants to optimize vaccine updates and preparedness strategies.

Here, we showed that *FluEmbed* establishes a robust approach for predicting influenza antigenicity from hemagglutinin (HA1) sequences by harnessing the ESM protein language model [23,31]. The model achieved strong performance in reproducing HI measurements, with predictions falling within a two-fold dilution of the observed values, which is consistent with other computational methods [8,11,27]. Importantly, *FluEmbed* outperformed all tested baselines, including classical sequence-distance measures (Hamming, BLOSUM62, Grantham) and the phylogenetic tree-based approach of Neher et al. (2016). The improvement over the Neher model is particularly noteworthy because the latter requires a pre-computed phylogenetic tree that, in turn, requires a multiple sequence alignment. However, *FluEmbed* operates directly on individual sequences, eliminating the computational bottleneck and potential alignment artifacts that can propagate into downstream analyses, particularly as sequence divergence and dataset size increase. The protein language model embeddings capture antigenic information that is not fully accessible through evolutionary distance on a phylogenetic tree, while simultaneously eliminating the alignment dependency that can limit scalability and introduce artifacts in large-scale surveillance applications. Recent studies have further advanced computational antigenicity prediction. Shah *et al.* (2024) developed a seasonal AdaBoost framework using amino acid mutation matrices and metadata to predict normalized HI titers, achieving an MAE of 0.702 under strict seasonal validation [12]. Jia *et al*. (2024) proposed *MetaFluAD*, combining graph neural networks with meta-learning to predict antigenic distances across multiple influenza subtypes [32]. While direct numerical comparison with these methods is not straightforward due to differences in datasets, antigenic distance definitions, and evaluation frameworks, *FluEmbed* offers a complementary approach that uniquely combines alignment-free protein language model embeddings with in silico mutagenesis to both predict antigenicity and characterize the functional constraints shaping antigenic evolution. We demonstrated that *FluEmbed* accurately captured meaningful antigenic signals compared to a null model. Furthermore, the model identified key immunodominant residues driving antigenic drift, many of which have been previously characterized [11,26,33], as well as new uncharacterised residues that may be pertinent for further study. Because *FluEmbed* is assay agnostic and could potentially integrate inhibition assays from multiple methods, for further validation, future studies could integrate in vitro HI assays and other microneutralization tests to compare predicted antigenic distances with measured serological responses. Reverse genetics [34] approaches can be used to generate influenza viruses carrying predicted high-impact mutations, allowing the direct assessment of their antigenic properties. Additionally, testing *FluEmbed’s* predictions against emerging strains from the WHO GISRS database could provide real-time validation and inform vaccine strain selection.

Temporal analysis revealed heterogeneity in predictive accuracy, presumably driven by antigenic evolution, with optimal predictive performance on time-matched train-test splits and declining accuracy on chronologically distant data. This finding highlights the importance of period-specific substitutions in shaping the evolutionary trajectory [35,36]. Our in silico mutational scanning highlights a key trade-off: conserved mutations (functionally constrained) generally yield smaller antigenic changes (limiting immune escape but preserving vital function), whereas more flexible unconstrained mutations usually have minimal antigenic effect but occasionally produce outlier variants with major immune escape. These results underscore how influenza’s immune escape dynamics are shaped by underlying functional constraints, as the virus typically ‘stays within the lines’ but has rare opportunities for larger antigenic jumps when exploring all possible amino acid mutations. Thus, *FluEmbed* can identify high-risk mutations that may facilitate immune escape. These predictions have been experimentally validated to confirm their antigenic effects [4]. Constrained variants exhibit a limited capacity to explore the antigenic space without disrupting critical attributes, as reflected in hemagglutination inhibition (HI) titers. This aligns with the extended evolutionary synthesis (EES), a paradigm that emphasizes that evolutionary trajectories are jointly shaped by selection pressures and developmental constraints [37]. Functional constraints governing antigenic evolution create a predictable landscape that offers the opportunity for rational vaccine design. Vaccine strategies can exploit these evolutionary limits by targeting conserved regions and minimizing escape mutations, while maintaining protection against circulating strains. These insights can inform vaccine strain selection by characterizing the antigenic landscape of evolving viral populations [38,39].

The lack of a significant difference between constrained and unconstrained mutations in 3C.2a suggests that antigenic drift within this lineage primarily occurs through intra-clade evolution rather than through distinct antigenic groups driven by competition for susceptible hosts. This highlights a shift from inter-lineage competition, where different viral clades vie for access to partially immune hosts, to more subtle within-clade changes that gradually modulate antigenicity. Understanding this pattern is crucial for vaccine strain selection, suggesting that updates may need to account for subtle intra-clade changes, rather than broad lineage replacements. By directly comparing *FluEmbed*’s antigenic drift predictions with WHO-selected vaccine strains, future studies will assess its potential for real-time influenza surveillance and vaccine strain selection, thereby advancing computational approaches for prospective antigenic forecasting.

The primary current limitations of *FluEmbed* are as follows: 1) Fine-tuning requires substantial computational resources, which necessitates high-performance hardware [40], although regularly fine-tuned pre-trained models (e.g., fine-tuning only a subset of parameters in ESM) for antigenicity prediction can reduce the computational costs. 2) Reliance on HI assay data may introduce bias due to assay limitations [41]. Incorporating data from alternative immunological assays, such as microneutralization tests [42] or flow cytometry-based antibody-binding assays [43], could provide a more comprehensive assessment of antigenic drift. 3) The effective antigenic distance between strains can vary, as prior exposure may confer transient cross-protection that dampens perceived differences. Integration of host-specific immune data can refine these predictions [44]. Thus, future *FluEmbed* iterations may incorporate host-specific immune data, such as longitudinal cohort studies, vaccine trial data, or serological profiling, during pretraining to refine antigenic predictions. 4) Influenza viruses adapt to environmental pressures [45]. Incorporating meta-data such as passage history (e.g., egg versus cell adaptation) or serum potency could help capture these dynamics and further improve the predictive performance of *FluEmbed.* 5) The training data in this study span 1968–2011, which predates the emergence and diversification of 3C.2a and subsequent H3N2 lineages. While *FluEmbed*’s methodology is generalizable to any time period with available HI data, the current model has not been validated on post-2011 strains. We note that *FluEmbed* is a flexible framework that, by design, can be retrained on updated datasets as they become available, and we encourage the community to apply the publicly available codebase to newer data. The 3C.2a in silico mutagenesis analysis presented here should therefore be interpreted as a prospective prediction of the model’s utility rather than a retrospective validation on directly observed 3C.2a-era titer data.

We also suggest that future studies explore eco-evolutionary constraints on influenza sequences by quantifying the variability in the predicted titer-distance change rates across lineages to quantify antigenic drift and shift dynamics [46]. Quantifying model precision and robustness through retrospective vaccine efficacy analysis and iterative refinement in wet laboratories is crucial. *FluEmbed's* approach can guide evolutionarily informed vaccine design by identifying antigens that elicit broad protective effects, minimizing the titer-distance between the current and future strains [47]. Thus, this approach could contribute to proactive data-driven influenza vaccine designs that take into account both past and future trajectories of virus evolution.

In addition, exploring the functional balance between influenza hemagglutinin (HA) and neuraminidase (NA) is a promising direction for future studies to refine the predictive performance of *FluEmbed.* The HA/NA balance, which reflects the equilibrium between HA binding affinity and NA enzymatic activity, critically affects viral fitness, host adaptation, and evolution [48,49]. Integrating computational approaches to quantify the HA/NA balance from sequence data into *FluEmbed* could provide additional signals for antigenic evolution and vaccine matching but would require further model development and validation.

While *FluEmbed* was developed specifically for influenza A(H3N2), the underlying protein language model approach may be generalizable to other antigenically variable pathogens, such as SARS-CoV-2 and respiratory syncytial virus (RSV). Given the success of AI-based modelling in characterizing SARS-CoV-2 immune escape mutations [50], future studies should evaluate whether *FluEmbed* embeddings capture antigenic drift in coronaviruses or other rapidly evolving RNA viruses, thereby contributing to broader infectious disease surveillance.

In conclusion, *FluEmbed* advances computational influenza surveillance and can accelerate vaccine design. For instance, the Centers for Disease Control and Prevention (CDC) could integrate *FluEmbed* into their vaccine strain selection process, even if only as a supplement to experimental assays. Integrating protein language models, interpretable learning, and in silico mutagenesis enables rapid characterization of antigenic phenotypes and real-time assessment of emerging subclades. While challenges remain, *FluEmbed*’s framework supports predictive infectious disease modelling and antigenic drift forecasting. Thus, alongside existing surveillance tools, *FluEmbed* could enhance our understanding of influenza evolution and contribute to the development of more effective evolutionarily informed vaccines.

## Materials and methods

### Datasets

Two datasets are used in this study. The influenza A virus data used for training and testing *FluEmbed* was originally compiled and published by Neher et al. (2016) [8]. It contains 8,599 antiserum-virus test pairs and their corresponding HI titer measurements. The accession number for each virus was used to extract HA1 amino acid sequences from the Global Initiative on Sharing Avian Influenza Data (GISAID) [51]. The data used to investigate *the in silico* mutagenesis of influenza A(H3N2) were obtained from historically observed subclades undergoing diversification and parallel evolution [52].

The raw HI titer values were transformed by first calculating the ${log}_{\text{2}\;}$ titers to normalize the distribution. Test-antiserum pairs with multiple HI measurements were averaged to obtain a single ${log}_{\text{2}}$ titer value. The HI titers were then standardized for each antiserum using the homologous titer method described by Neher *et al.* (2016) [8]. Briefly, the homologous titer of an antiserum was defined as the HI titer against a virus strain identical to that of the antiserum. If no identical homologous viruses were present, the maximum HI titer was used. The standardized HI was calculated by subtracting the ${log}_{\text{2}}$ titer from the homologous ${log}_{\text{2}}$ titer.

To develop and validate *FluEmbed*, we partitioned the dataset into training (90%) and testing (10%) sets. This distribution replicated the real-world prediction task, with *FluEmbed* encountering novel influenza strains that were absent during training. We employed a sampling strategy (S1 Appendix, Text A) to exclude unique viral strains from the test set. In alignment with standard wet lab assays, each antiserum must be pre-established and incorporated within the training phase of the model. This ensures that *FluEmbed* maintains a foundational knowledge base to effectively assess and predict the behavior of new viral mutations. The resulting partition yielded 5,750 training pairs (~96.31%) and 220 test pairs (~3.69%) at the assay level, corresponding to 355 training viruses and 22 test viruses at the strain level (S1 Appendix, Fig A). An additional 25 non-reference strains (including post-2011 isolates) were held as an independent reserve set for future validation.

### Sequence to embedding representation

An Evolutionary Scale Model with 33 layers, 650 million parameters, and an output layer of 1280 dimensions at each amino acid residue (esm2_t33_650M_UR50D) [23,31] was used to generate embedding representations for each Hemagglutinin (HA) protein sequence. In contrast to simplistic measures such as sequence similarity, ESM fixed-length vector embeddings capture complex contextual and structural constraints [23]. We evaluated the performance of the ESM models esm2_t36_ 3 B _UR50D, esm2_t48_ 15 B _UR50D, and esm2_t33_650M_UR50D before selecting esm2_t33_650M_UR50D for optimal performance at a comparatively low computational cost. Because the HA1 protein has only 329 amino acid residues, a larger ESM model could be considered excessive for embedding representations [22]. For each sequence, residue embeddings were averaged to produce a fixed 1280-dimensional vector representation of the entire sequence, which was invariant to protein length (S1 Appendix, Text A). Mean pooling was chosen to capture full contextual information across the HA1 sequence, including non-epitope regions that may contribute to antigenic properties through long-range structural interactions, consistent with best practices for protein language model applications [23]. Sensitivity analysis comparing mean pooling with CLS token pooling confirmed that error patterns are robust to pooling strategy choice (S2 Appendix, Section 7).

### Embedding to HI titer prediction

For each antiserum-virus pair, the absolute value of the vector difference between the HA protein embeddings created a 1280-dimensional vector representation of the pair. This pair-difference embedding vector was used to predict the standardized HI titer. To map the embedding signal to the standardized HI titer, we used a random forest model [53]. The models were optimized through a 10-fold cross-validation of the training data. Hyperparameter tuning uses a Bayesian optimization algorithm [54]. The optimized hyperparameters that resulted in the lowest cross-validation errors were selected (S1 Appendix, Text B). The process involved optimizing these random forest model hyperparameters: number of trees (n_estimators) from 100 to 500, maximum tree depth (max_depth) from 10 to 100, minimum samples to split an internal node (min_samples_split) from 2 to 10, minimum samples at a leaf node (min_samples_leaf) from 1 to 4, and maximum features for splitting (max_features) from 0.1 to 0.999. Additionally, the models were trained on different temporal partitions of the dataset to assess *FluEmbed’*s ability to generalize across antigenic drift epochs.

The performance of *FluEmbed* was evaluated on the held-out test set using metrics, such as the Spearman correlation coefficient, MAE, and RMSE. The Spearman correlation coefficient is a non-parametric metric that measures the rank-order similarity between predicted and experimental antigenic distances. MAE and RMSE were also computed to further confirm the robustness of our framework, given that antigenic drift is best assessed in a relative rather than absolute manner because absolute HI titers can be influenced by assay conditions, baseline variability, and other external factors [55]. We estimated the confidence intervals for the RMSE and MAE as the mean ± standard error. To further assess the differences in model performance, a two-sample t-test was used to compare the MAE and RMSE distributions between the trained and null models. The resulting *p*-values quantify the probability that the observed differences in the MAE and RMSE distributions between the trained and null models could have occurred through random chance (S1 Appendix, Text B).

### Biological inference and residue importance

To investigate the biological importance of specific amino acid residues in influenza antigenicity, in silico deletion experiments were performed (S1 Appendix; Text C). For each antiserum-virus pair, we systematically deleted the residue(s) in both sequences. The resulting sequences with deletions were converted into embeddings using ESM. These embeddings were used to predict the HI titers using the trained *FluEmbed* model. The mean absolute error (MAE) between the predicted and actual HI titers was calculated for each deletion mutant. The difference in MAE between the deletion mutant and original sequence was computed for each residue position. This difference quantifies the impact of each residue on the *FluEmbed*'s predictive performance, serving as a proxy for the residue's importance in shaping antigenic properties.

To summarize the importance of each residue position across multiple antiserum-virus pairs, we calculated the average MAE difference. The resulting average MAE differences per residue position were used to identify key residues contributing to influenza antigenicity. The top 2.5% of the residues were highlighted as potentially important antigenic determinants. The biological relevance of the residues was validated by comparing them with known antigenic sites reported in the literature. Open black triangles were assigned to residues previously identified in the literature, and red triangles were assigned to residues that persisted throughout the experimental period. Residues marked with red triangles and black outlines represent sites that persisted throughout the experimental period and were previously identified in the literature.

### Viral subclade *in Silico* mutagenesis

To elucidate the mechanisms of viral evolution and antigenic variability, we developed a probabilistic in silico mutagenesis approach to explore potential mutations in influenza A virus hemagglutinin (HA) protein sequences, focusing on constrained and unconstrained mutation frameworks. Mutation frequencies and position rankings were derived from *Nextclade* (https://clades.nextstrain.org), which provides historical amino acid frequency data across globally circulating H3N2 lineages. The consensus sequences, position-specific amino acid frequencies, and position importance rankings for each subclade are provided in S1 Appendix (A–C Tables, Text E). To convert *FluEmbed*’s standardized HI predictions to predicted HI titers (Fig 6), we applied the inverse standardization using the homologous titer of each reference antiserum (S1 Appendix, Text E). Statistical comparisons between constrained and unconstrained mutation distributions were performed using the Wilcoxon rank-sum test with Benjamini-Hochberg FDR correction for multiple comparisons.

Constrained variants were generated by selecting the top K positions ($p_{\text{1}}$, …, $p_{K}$) most prone to mutation based on historical mutation frequency data from *Nextclade* (https://clades.nextstrain.org) and considering the N most probable amino acid substitutions at each position [52]. This reduced the theoretical mutation space from ${\text{2}\text{0}}^{K}$ to ${\left(N-p\right)}^{K}$, where p represents positions where the original amino acid remains among the N most probable mutations, ensuring biological relevance while maintaining computational feasibility. For unconstrained variants, all 20 possible amino acids were considered at each of the K positions, allowing exhaustive exploration of the mutational landscape. Using the consensus sequence of each clade as a template, we generated mutant sequences and predicted their antigenic effects using *FluEmbed* (S1 Appendix, Text D). Scatter plots (S1 Appendix, Figs B and C) displayed possible constrained and unconstrained mutational outcomes for A/Perth/16/2009 and A/Victoria/361/2011, illustrating expanded antigenic variability in unconstrained mutations. This framework enables the identification of key residues that balance immune escape with functional viability and informs the selection of vaccine strains.

## Supporting information

S1 Appendix Supplementary methods, algorithms, tables (A–C Tables), and figures (A–C Figs) for FluEmbed. Includes data preprocessing algorithms, model training and validation procedures, biological inference methods, in silico mutagenesis framework, Nextclade-derived mutation data, and titer transformation procedures.
(DOCX)

S2 Appendix Sensitivity analyses and baseline comparisons for FluEmbed. Includes reproducibility validation, residue importance method comparison, random seed stability, stratified sampling evaluation, embedding and sequence-distance baseline comparisons, CLS token pooling analysis, and Neher et al. (2016) tree model comparison.
(DOCX)
