# Supplementary material for: Alignment-free prediction of cross-reactivity in influenza A (H3N2) anticipates antigenic drift
Source: PLoS Comput Biol. 2026 Aug 11;22(8):e1014628. doi: 10.1371/journal.pcbi.1014628 (PMC13497264; doi:10.1371/journal.pcbi.1014628)
Supplement: Supplementary file 1 [file pcbi.1014628.s001.docx]

**Supporting information 1 (SI 1) for**

**Alignment-Free Prediction of Cross-Reactivity in Influenza A (H3N2) Anticipates Antigenic Drift**

**Authors**: Alpha Forna^a,b,f*^, Lambodhar Damodaran^c,h^, Christian E. Gunning^a,b^, Parnian Rahimi ^e,h^, Aarya Venkat^h^, Natarajan Kannan^h^, Rebecca Kondor^g^, Justin Bahl^b,e^, Pejman Rohani^a,b,d,e,#^, John M. Drake^a,b,e,h,#^

**Affiliations**:

^a^Odum School of Ecology, University of Georgia, Athens, Georgia, USA.

^b^Center for the Ecology of Infectious Diseases, University of Georgia, Athens, Georgia, USA.

^c^Department of Pathobiology, School of Veterinary Medicine, University of Pennsylvania, Philadelphia, Pennsylvania, USA.

^d^Department of Infectious Diseases, University of Georgia, Athens, Georgia, USA.

^e^Center for Influenza Disease & Emergence Research (CIDER), Athens, Georgia, USA

^f^Department of Epidemiology and Biostatistics, College of Public Health, University of Georgia, Athens, Georgia, USA

^g^Centers for Disease Control and Prevention, Atlanta, Georgia, USA.

^h^Institute of Bioinformatics, University of Georgia, Athens, Georgia, USA.

^#^These authors contributed equally to this work.

*[alpha.forna@uga.edu](mailto:alpha.forna@uga.edu)

**SUPPLEMENTARY INFORMATION TEXT**

**Text A**

1. **Data Preprocessing and Feature Construction**

**Algorithm 1: Data Partitioning**

1. Load the dataset

2. Filter out instances with missing values.

3. Validate Protein Sequences:

- Ensure sequences only contain valid amino acid characters.

4. Calculate HA1 Protein Length:

- For each protein sequence, calculate its length.

5. Filter by Length:

- Filter data based on sequence length (>= 566).

6. Extract the HA1 Regions:

- Extract regions of interest from the sequences (e.g., positions 17 to 345, 329 amino acid residues).

**Algorithm 2: Embedding Generation**

1. Use ESM-2 (we use the 650M parameter) Model:

- Load the ESM-2 model for embedding generation.

2. Generate Sequence Embeddings:

- For each sequence, generate embeddings by averaging token embeddings.

3. Difference between Embeddings:

- Difference between antiserum and virus embeddings form joint representations.

**Algorithm 3: Sampling Strategies**

1. Randomly Sample All Viral Antigenic Clusters:

1. Exclude Specific Viruses:

- Exclude specific viruses from the antiserum strains.

2. Split Data:

- Split data accordingly into training (90%) and testing sets (10%).

**Text B**

2. **Model Training and Validation**

**Algorithm 4: Model Training**

1. Load Training Data:

- Load the pre-processed training data from the pickle file.

2. Create Difference Variable (if not done already in preprocessing):

- Compute the difference between Serum and Virus embeddings.

3. Extract Features and Labels:

- Extract the difference embeddings as features $X_{train}$.

- Extract standardized HI titers as labels $y_{train}$.

4. Train Model:

1. Define Cross-Validation Function for Random Forest:

- Initialize a Random Forest Regressor with hyperparameters.

- Perform cross-validation on training data.

- Return mean squared error (MSE) scores.

2. Bayesian Optimization for Hyperparameter Tuning:

- Define the parameter bounds.

- Maximize the cross-validation function.

- Retrieve the best hyperparameters.

3. Train Final Model:

- Train the Random Forest Regressor using the best hyperparameters on the entire training data.

4. Save the Model:

- Save the trained model to a file.

**Algorithm 5: Model Validation**

1. Load Model and Data:

- Load the trained random forest model from the file.

- Load the pre-processed testing data from the file.

2. Extract Features and Labels:

- Extract the difference embeddings as features $X_{test}$.

- Extract standardized HI titers as labels $y_{test}$.

3. Evaluate Model Performance:

- Predict using the trained model on both training and testing data.

- Calculate evaluation metrics (RMSE, MAE, Spearman Correlation).

4. Compute Confidence Intervals:

- Compute confidence intervals for the evaluation metrics using standard errors.

5. Save Evaluation Metrics and Predictions:

- Save the evaluation metrics and confidence intervals to a CSV file.

- Save the actual and predicted values for both training and testing data to pickle files.

**Text C**

3. **Biological Inference**

**Algorithm 6: Biological Inference**

1. Load Data:

- Load the pre-processed training and testing data from the pickle files.

2. Initialize Metrics:

- Define the metrics to evaluate (e.g., MAE).

3. Iterate Through Each Virus/Antiserum Pair:

- For each pair in the testing data:

1. Extract virus and antiserum sequences.

2. Generate embeddings for the sequences.

3. Make initial predictions and calculate baseline metrics.

4. Mutate and Evaluate Residues:

- For each residue position (1 to 329):

1. Generate mutant sequences by deleting the residue.

2. Generate embeddings for the mutant sequences.

3. Make predictions with the mutant embeddings.

4. Calculate metrics for mutant predictions.

5. Compare mutant metrics with baseline metrics and track differences.

5. Average Metrics:

- Calculate the average metrics for each residue position.

- Sort and save the results.

**Text D**

4. **Viral Subclade *in Silico* Mutagenesis**

**Algorithm 7: Probabilistic In Silico Mutagenesis Analysis**

1. Data Loading and Preparation:

- Load consensus sequences for each clade

- Import historical mutation frequency data from Nextclade

- Load FluEmbed model and preprocessed data

2. Mutation Space Definition:

A. Constrained Framework:

- Identify top K positions from frequency data

- Extract N most probable amino acids per position

- Calculate reduced mutation space (N-p)^K

B. Unconstrained Framework:

- Use same K positions

- Consider all 20 amino acids

- Mutation space = 20^K

3. Sequence Generation:

For each clade consensus sequence:

- Generate constrained mutations using probability-filtered substitutions

- Generate unconstrained mutations using all amino acids

- Random sample 1000 sequences from each framework if computationally necessary

4. Antigenic Impact Prediction:

For each mutant sequence:

- Generate sequence embeddings using FluEmbed

- Calculate difference embeddings against reference antigens

- Predict antigenic distances

- Record predictions and mutation details

5. Analysis and Visualization:

- Calculate average antigenic impact per mutation

- Generate scatter plots of antigenic outcomes

- Compare constrained vs unconstrained frameworks

- Identify key antigenic-modulating residues

6. Output and Interpretation:

- Save mutation-impact mappings

- Document key positions balancing immune escape and viability

- Generate visualizations of antigenic landscapes

## Text E

**5. In Silico Mutagenesis Data Sources and Titer Transformation**

The in silico mutagenesis analysis (Fig 6) used three data files derived from Nextclade (https://clades.nextstrain.org ) to define the mutation space for each H3N2 subclade:

(i) Clade consensus sequences (Table A): For each subclade (3B, 3C, 3C.2, 3C.2a, 3C.3, 3C.3a, 3C.3b), the consensus HA amino acid sequence was obtained from Nextclade’s clade definitions. The HA1 region (positions 17–344 of the full HA protein, yielding 328 residues) was extracted for embedding and prediction.

(ii) Position-specific amino acid frequencies (Table B): Historical mutation frequency data from Nextclade were used to compute the probability of each amino acid at each HA1 position. For the constrained mutagenesis framework, only the top 5 most probable amino acids at each position were considered, restricting the mutation space to biologically plausible substitutions. For the unconstrained framework, all 20 amino acids were permitted at each position.

(iii) Position importance rankings (Table C): For each subclade, HA1 positions were ranked by their historical mutation frequency. The top 5 most mutation-prone positions per subclade were selected as the sites for in silico mutagenesis.

**Titer Transformation.** The FluEmbed model predicts standardized HI titers (log^₂^-scale difference from the homologous titer). To convert these predictions to predicted HI titers (as shown in Fig 6), we applied the inverse of the standardization: titre_log_2_ = homologous_log − standardized_HI, followed by titre = 2^titre_log₂^. The homologous log_2_ titer for each antiserum was calculated as the mean log^₂^ titer of the antiserum against its homologous virus strain in the training data. For the A/Perth/16/2009 antiserum, the homologous titer was derived from training pairs where serumClusters = ‘PE09’ and the virus matched the serum strain. The same procedure was applied for A/Victoria/361/2011 (serumClusters = ‘SO11’). The 3-fold dilution threshold shown in Fig 6 represents the predicted titer value at one-third of the homologous titer, a standard threshold for assessing meaningful antigenic differences.

**Statistical Comparisons.** Constrained and unconstrained predicted titer distributions were compared within each subclade using the Wilcoxon rank-sum test. All p-values were corrected for multiple comparisons using the Benjamini-Hochberg false discovery rate (FDR) procedure across all subclades within each panel of Fig 6.

These supplementary data files (Tables A–C) are provided for full transparency and reproducibility of the in silico mutagenesis analysis.

**Table A:** *clade_consensus_nextclade.csv* — Nextclade-derived consensus HA amino acid sequences for each H3N2 subclade (3B, 3C, 3C.2, 3C.2a, 3C.3, 3C.3a, 3C.3b). The HA1 region (positions 17–344) was extracted for embedding and prediction.

**Table B:** *clade.pos.aa_updated.csv* — Nextclade-derived amino acid frequency data at each HA1 position. Contains columns: position, amino acid, count (N). Used to compute the probability of each amino acid at each position for the constrained mutagenesis framework.

**Table C:** *clade.pos.rank_updated.csv* — Nextclade-derived position importance rankings for each subclade. Contains columns: clade, position, position rank. The top 5 most mutation-prone positions per subclade were selected as mutagenesis targets.

**SUPPLEMENTARY TABLES AND FIGURES**

**SUPPLEMENTARY TABLES**

Table A: Consensus Amino Acid Sequences per H3N2 Subclade

Consensus HA1 amino acid sequences (329 residues, positions 17–345 of the full HA protein) for each H3N2 subclade, derived from Nextclade (https://clades.nextstrain.org). These sequences serve as the starting templates for the constrained and unconstrained in-silico mutagenesis frameworks described in the main text. Full sequences are provided in the accompanying CSV file (clade_consensus_nextclade.csv), available at: <https://doi.org/10.17605/OSF.IO/JKF5B>.

| Clade | Consensus Sequence (HA1, 329 AA) | Source |
| --- | --- | --- |
| 3B | Clade 3B consensus | Derived from Nextclade alignment of circulating 3B strains |
| 3C | Clade 3C consensus | Derived from Nextclade alignment of circulating 3C strains |
| 3C.2 | Clade 3C.2 consensus | Derived from Nextclade alignment of circulating 3C.2 strains |
| 3C.2a | Clade 3C.2a consensus | Derived from Nextclade alignment of circulating 3C.2a strains |
| 3C.3 | Clade 3C.3 consensus | Derived from Nextclade alignment of circulating 3C.3 strains |
| 3C.3a | Clade 3C.3a consensus | Derived from Nextclade alignment of circulating 3C.3a strains |
| 3C.3b | Clade 3C.3b consensus | Derived from Nextclade alignment of circulating 3C.3b strains |

*Note: Full-length consensus sequences (329 AA each) are provided in the CSV file. Only summary information is shown here. The complete file is available at:* [*https://doi.org/10.17605/OSF.IO/JKF5B*](https://doi.org/10.17605/OSF.IO/JKF5B)*.*

Table B: Amino Acid Frequencies at Each HA1 Position per Subclade

Amino acid frequencies at each of the 329 HA1 positions for each H3N2 subclade, derived from historical sequence data via Nextclade. These frequencies define the constrained mutagenesis framework: for each position, only the N most probable amino acid substitutions (based on observed frequencies) are considered as candidate mutations. Positions where the consensus amino acid remains among the top N most probable are excluded from the constrained mutation space. Representative rows are shown below; the complete dataset is provided in clade.pos.aa_updated.csv available at : <https://doi.org/10.17605/OSF.IO/JKF5B>.

| HA1 Position | Amino Acid | Frequency | Clade |
| --- | --- | --- | --- |
| 1 | Q | 0.87 | 3B |
| 1 | R | 0.08 | 3B |
| 1 | K | 0.03 | 3B |
| 2 | D | 0.92 | 3B |
| 2 | N | 0.05 | 3B |
| 156 | H | 0.45 | 3C.2a |
| 156 | S | 0.31 | 3C.2a |
| 156 | N | 0.12 | 3C.2a |
| 159 | Y | 0.52 | 3C.2a |
| 159 | F | 0.28 | 3C.2a |
| ... | ... | ... | ... |

*Note: Table truncated for display. The full file contains amino acid frequencies for all 329 positions across all 7 subclades (3B, 3C, 3C.2, 3C.2a, 3C.3, 3C.3a, 3C.3b). Available at:* [*https://doi.org/10.17605/OSF.IO/JKF5B*](https://doi.org/10.17605/OSF.IO/JKF5B)*.*

Table C: Position Importance Rankings per Subclade

Position importance rankings for each H3N2 subclade, derived from historical mutation frequency data via Nextclade. Rankings identify the top K positions most prone to mutation within each clade. These rankings determine which positions are selected for the in-silico mutagenesis scanning (both constrained and unconstrained frameworks). Higher-ranked positions (rank = 1 being most important) have the highest historical mutation frequencies within their respective subclade. Known antigenic site annotations are included where applicable. Representative rows are shown below; the complete dataset is provided in clade.pos.rank_updated.csv available at: <https://doi.org/10.17605/OSF.IO/JKF5B>.

| Clade | HA1 Position | Rank | Known Antigenic Site |
| --- | --- | --- | --- |
| 3B | 156 | 1 | Antigenic site B |
| 3B | 159 | 2 | Antigenic site B |
| 3B | 189 | 3 | Antigenic site B |
| 3B | 145 | 4 | Antigenic site A |
| 3B | 193 | 5 | Antigenic site B |
| 3C.2a | 159 | 1 | Antigenic site B |
| 3C.2a | 156 | 2 | Antigenic site B |
| 3C.2a | 160 | 3 | Near antigenic site B |
| 3C.2a | 145 | 4 | Antigenic site A |
| 3C.2a | 193 | 5 | Antigenic site B |
| ... | ... | ... | ... |

*Note: Table truncated for display. The full file contains position rankings for all 329 HA1 positions across all 7 subclades. Available at:* [*https://doi.org/10.17605/OSF.IO/JKF5B*](https://doi.org/10.17605/OSF.IO/JKF5B)*.*

Fig A: Data processing and partitioning workflow for the FluEmbed model. (A) Initial dataset of HI assays with 8599 rows. (B) Data filtering process: removal of rows with blank AA sequences, titer ≤ 40, and standard titer < -2; calculation of log2 titer and standard titer; averaging of log2 titer for unique serum-virus pairs, resulting in 6197 filtered rows. (C) Sampling 5970 rows (~96.34% of filtered data) for model development. (D) Further division into training (5750 rows, ~96.31% of samples) and validation (220 rows, ~3.69% of samples) sets. (E) Identification of unique virus strains in the dataset: 402 unique viruses, including 152 reference viruses and 250 non-reference viruses. (F) Partitioning of unique viruses: 355 for training (including all 152 reference viruses) and 22 for validation (10% of non-reference viruses). (G) Reserve sets maintained at both the HI assay level (227 rows) and the unique virus level (25 non-reference viruses). This workflow ensures a robust dataset for model training and unbiased validation, while preserving a portion of the data for potential future testing.

Fig B: Predicted HI titers for constrained (circles) and unconstrained (triangles) variants of A/Perth/16/2009 across H3N2 subclades. Constrained variants show tighter antigenic distributions, while unconstrained variants explore broader mutational space, reflecting enhanced immune escape potential.

Fig C: Predicted HI titers for constrained (circles) and unconstrained (triangles) variants of A/Perth/16/2009 across H3N2 subclades. Constrained variants show tighter antigenic distributions, while unconstrained variants explore broader mutational space, reflecting enhanced immune escape potential.
