# Supplementary material for: Alignment-free prediction of cross-reactivity in influenza A (H3N2) anticipates antigenic drift
Source: PLoS Comput Biol. 2026 Aug 11;22(8):e1014628. doi: 10.1371/journal.pcbi.1014628 (PMC13497264; doi:10.1371/journal.pcbi.1014628)
Supplement: Supplementary file 2 [file pcbi.1014628.s002.docx]

**Supporting information 2 (SI 2) for**

**Alignment-Free Prediction of Cross-Reactivity in Influenza A (H3N2) Anticipates Antigenic Drift**

**Authors**: Alpha Forna^a,b,f*^, Lambodhar Damodaran^c,h^, Christian E. Gunning^a,b^, Parnian Rahimi ^e,h^, Aarya Venkat^h^, Natarajan Kannan^h^, Rebecca Kondor^g^, Justin Bahl^b,e^, Pejman Rohani^a,b,d,e,#^, John M. Drake^a,b,e,h,#^

**Affiliations**:

^a^Odum School of Ecology, University of Georgia, Athens, Georgia, USA.

^b^Center for the Ecology of Infectious Diseases, University of Georgia, Athens, Georgia, USA.

^c^Department of Pathobiology, School of Veterinary Medicine, University of Pennsylvania, Philadelphia, Pennsylvania, USA.

^d^Department of Infectious Diseases, University of Georgia, Athens, Georgia, USA.

^e^Center for Influenza Disease & Emergence Research (CIDER), Athens, Georgia, USA .

^f^Department of Epidemiology and Biostatistics, College of Public Health, University of Georgia, Athens, Georgia, USA

^g^Centers for Disease Control and Prevention, Atlanta, Georgia, USA.

^h^Institute of Bioinformatics, University of Georgia, Athens, Georgia, USA.

^#^These authors contributed equally to this work.

* [alpha.forna@uga.edu](mailto:alpha.forna@uga.edu)

**Summary of Analyses and Key Insights**

**1. Reproducibility of the Influenza Embedding Pipeline**

The goal of this analysis was to evaluate the reproducibility and stability of our influenza embedding pipeline. To do this, we re-ran the full workflow using the same parameters and configurations as in the original study and then compared the results both across random seeds and against the paper’s reported findings. Figs A and B show the reproducibility tests, where the random forest was re-trained using identical settings to the original pipeline.

Fig A shows the cross-period performance heatmaps, comparing mean absolute error (MAE) and Spearman correlation between training and testing periods. The results confirm that the model captures consistent temporal relationships—errors remain stable along the diagonal, indicating reliable within-period performance.

Fig B presents the amino acid position importance across time periods. The persistence of key HA1 positions among the top-ranked sites demonstrates that the model identifies biologically relevant antigenic regions, consistent with known influenza important sites.

Fig A

Fig B

Figs C, D, and E compare the results from this re-run against the original paper’s reported errors.

- Fig C shows direct error comparisons across all time periods, revealing near-perfect alignment between reproduced and reported MAE values, with most points clustering tightly around the 1:1 diagonal.
- Fig D visualizes the density of error differences, showing that discrepancies between these results and in the manuscript are centered near zero, with minimal spread indicating numerical consistency between runs.
- Figs D and F apply log₁₀ transformations and square-root to test whether scaling differences affect comparability. Even under these transformations, the agreement remains nearly exact, confirming that both pipelines behave identically across different value ranges and transformations.

Overall, the results demonstrate that the influenza embedding pipeline is reproducible. The consistency across time periods, the preservation of biologically meaningful position signals, and the minimal error deviation relative to the original study all reinforce the stability, reliability, and interpretability of the modeling framework.

Fig C

Fig D

Fig E

Fig F

**2. Evaluation of Residue Importance Methods: Dash vs. Mask Approaches**

Figs G to J evaluate the residue importance calculation methods, where the original study used a “dash” approach, while the reproduced pipeline employed a “mask” technique for feature masking. The strong correspondence in MAE patterns (seen in scatter and histogram comparisons) indicates that replacing the dash-based masking with the mask-based method does not alter the results. This confirms that residue-level importance is an inherent property of the model and not sensitive to the specific masking implementation.

In addition, to identify which amino acid positions contribute most strongly to model performance, we employed a mask-based importance analysis using the ESM2 transformer model. For each HA1 position, we systematically masked the amino acid and measured how much this masking increased the model's prediction error. Positions that cause larger increases in error are interpreted as more influential for the model’s ability to make accurate predictions. Fig K shows the average MAE increase per position across multiple time periods. Each subplot corresponds to a distinct evolutionary period, allowing us to examine how positional importance shifts through time.

Fig G

Fig H

Fig I

Fig J

Fig K

**3. Sensitivity of Random Forest Performance to Random Seed Choice**

To examine the impact of random seed choice on model performance within our Random Forest, we compared the error metrics from four alternative seeds against the commonly used baseline seed of 42. Each subplot represents a specific period, showing the average MAE error for Seed 42 (y-axis) plotted against the corresponding error from another seed (x-axis). Points lying near the red dashed 45° line indicate similar performance between Seed 42 and the comparison seed, whereas deviations from the line highlight periods or parameter settings where seed choice influences model outcomes. Overall, the plots allow us to visually assess the stability and robustness of the Random Forest model with respect to varying random seeds across multiple time windows.

Fig L

After observing that certain periods in our seed comparison analysis appeared more sensitive to changes in the Random Forest random seed, we investigated whether differences in the underlying data distribution could be contributing to this effect. Fig M shows the training and testing sample distributions for each time period, with blue bars representing training samples and red bars showing testing samples. Each subplot corresponds to one period used in our pipeline and displays the number of samples available in each year within that window. Periods with uneven distributions, limited sample counts, or years dominated by testing data may introduce additional variability in model performance, making them more susceptible to fluctuations caused by different random seeds.

By examining these distributions, we can better understand why certain periods exhibit stronger seed-dependent behavior in the earlier figure.

Fig M

**4. Improving Train–Test Balance Using Stratified Sampling**

Our earlier plots showed that some periods contained very few test samples. Such imbalances can cause instability in model evaluation, amplifying the effect of randomness during training. To address this issue, we applied stratified sampling within each period to ensure that the train–test split more accurately reflected the underlying year-by-year composition of the data. Fig N labeled displays the resulting distribution. Unlike the original configuration, each period now contains a more consistent and representative number of test samples, with both training and testing sets better aligned with the actual temporal distribution of observations. The final comparison plot (Fig O) highlights how dramatic this improvement is.

Overall, stratified sampling produced a more balanced, fair, and stable dataset for model training and evaluation, helping ensure that differences in model performance are due to model behavior rather than artifacts of imbalanced sampling.

Fig N

Fig O

**5. Effect of Stratified Sampling on Model Error Stability**

To evaluate whether the uneven and inconsistent test-sample distribution in the original setup contributed to instability in model performance, we compared the model errors obtained under the previous (non-stratified) split with those obtained under the new stratified split. The stratified approach produces a more balanced representation of each year within every train–test period, reducing the likelihood that certain periods are overly influenced by randomness or sparse data.

Fig O shows the side-by-side error comparison across all periods, with the original error on one axis and the new-split error on the other. Points near the 45° reference line indicate similar performance between the two splits, while points deviating from the line highlight periods where the choice of split has a larger influence. Some periods particularly those that originally had very few test samples show greater spread, suggesting that the earlier variability was due not only to random seed effects but also to structural issues in the data distribution.

To explore this further, we examined the same comparison under square-root and log-scaled transformations figs Q and R, which help normalize the heavy-tailed nature of the MAE values. Across these transformations, the general pattern remains consistent: most points cluster around parity, but a subset of configurations exhibit larger differences, consistent with noise fluctuations or inherent instability in those specific periods.

Finally, the histograms of error differences (Fig S) provide a distributional view of how much the new split changes the resulting performance. For most periods, the mean difference lies close to zero, indicating that the new distribution does not systematically inflate or reduce model error. However, in some periods the tails are heavier, reflecting occasional configurations where the original split produced unusually high or low errors, likely a consequence of data scarcity, year-level irregularities, or noisy underlying relationships.

Overall, these comparisons confirm that while the model remains broadly stable across both splitting strategies, certain periods are more sensitive to the structure of the data itself. Variability previously attributed to random seeds may, in part, stem from the nature of the data within those windows, reinforcing the need for balanced sampling such as the stratified approach used here.

Fig P

Fig Q

Fig R

Fig S

**6- Comparative Performance of Embedding and Sequence-Based Distances for Antigenic Prediction**

Our primary objective was to determine whether ESM-2 cosine embedding distance provides a more informative predictor of antigenic distance than traditional sequence based metrics, and secondarily, whether a refined version of Hamming distance genuinely improves performance. The results from all comparisons point to two main conclusions:

A) Sequence-based distances perform similarly and show minimal top-line differences.

Across Hamming, BLOSUM, and Grantham distances, the correlations with antigenic distance are remarkably consistent (Spearman ρ ≈ 0.55–0.56). Despite their conceptual differences, raw amino acid mismatches, substitution-constrained distances, or biochemical property weighting, all of these metrics capture antigenic change to roughly the same extent. This uniformity was somewhat surprising but empirically robust.

### B) Cosine embedding distance is noticeably less predictive.

ESM-2 cosine distance shows substantially weaker correlation with antigenic distance (ρ ≈ 0.41). The scatter exhibits much larger vertical noise and a less coherent trend than any of the sequence-based metrics. Although embeddings encode general biological structure, their raw cosine geometry does not appear to target antigenically relevant variation in this dataset.
 This result directly answers our primary question.

Fig T

Fig U

**7- CLS token**

We modified the embedding pooling strategy by replacing mean pooling with CLS token pooling in order to evaluate whether the CLS representation provides a more informative summary of sequence-level features. Then compared model errors derived from CLS pooling against previously reported paper errors across multiple historical time periods.

The error comparison plots show a positive correlation between CLS-based errors and paper-reported errors across all periods, with most points clustering near the diagonal. Overall, this indicates that changing the pooling strategy does not substantially alter error patterns across sites, while preserving consistency with prior benchmarks.

Fig V

**8- Comparison with the Neher et al. (2016) Phylogenetic Tree Model**

To benchmark FluEmbed against an established alignment-dependent method, we implemented the phylogenetic tree-based antigenic prediction model of Neher et al. (2016) using augur's TreeModel framework. This model decomposes the predicted titer as Ĥ_αβ = v_α + p_β + D_ab, where v_α represents virus avidity, p_β represents serum potency, and D_ab represents the antigenic distance accumulated along branches of the phylogenetic tree separating the virus and serum strains. The model was trained on the same 5,807 training pairs used for FluEmbed (Strategy 2 partition) and evaluated on the held-out test set. Tree construction required a pre-computed multiple sequence alignment and phylogenetic inference, representing a fundamentally different (i.e., alignment-dependent) approach to antigenic prediction.

On the held-out test set, the Neher tree model achieved a Spearman ρ of 0.654 (Pearson r = 0.654, RMSE = 1.779, MAE = 1.418), compared with FluEmbed's Spearman ρ of 0.727 (Pearson r = 0.728, RMSE = 1.299, MAE = 0.945). FluEmbed thus outperformed the Neher model by approximately 11% in rank correlation and achieved substantially lower prediction errors (27% reduction in RMSE, 33% reduction in MAE). Notably, the Neher model exhibited pronounced temporal bias in its residuals, with systematic overprediction for viruses isolated around 1989–1991 and underprediction for post-2000 strains (Fig Y), suggesting that the tree diffusion model's assumptions about uniform antigenic change along phylogenetic branches may not hold across all evolutionary epochs. These results demonstrate that FluEmbed's alignment-free protein language model embeddings capture antigenic information beyond what is accessible through phylogenetic distance alone, while simultaneously avoiding the computational requirements and potential artifacts of multiple sequence alignment and tree inference.

Fig W

Fig X

Fig Y
